# Lateral position during severe mono-lateral pneumonia: an experimental study

**DOI:** 10.1038/s41598-020-76216-w

**Published:** 2020-11-09

**Authors:** Andrea Meli, Enric Barbeta Viñas, Denise Battaglini, Gianluigi Li Bassi, Hua Yang, Minlan Yang, Joaquim Bobi, Ana Motos, Laia Fernández-Barat, Davide Chiumello, Paolo Pelosi, Antoni Torres

**Affiliations:** 1 Department of Pulmonology, Hospital Clínic of Barcelona, Barcelona, Spain; 2 University of Milan, Milan, Italy; 3 Department of Surgical Sciences and Integrated Diagnostics (DISC), IRCCS San Martino Policlinico Hospital, Genoa, Italy; 4 Centro de Investigación en Red de Enfermedades Respiratorias (CIBERES), Madrid, Spain; 5 Institut d’Investigacions Biomèdiques August Pi I Sunyer (IDIBAPS), Barcelona, Spain; 6 University of Barcelona (UB), Barcelona, Spain; 7 Department of Anaesthesia and Critical Care Medicine, ASST Santi Paolo e Carlo, San Paolo Hospital, Milan, Italy

**Keywords:** Ultrasonography, Infectious diseases, Respiratory tract diseases, Experimental models of disease, Medical research

## Abstract

Patients with mono-lateral pneumonia and severe respiratory failure can be positioned in lateral decubitus, with the healthy lung dependent, to improve ventilation-perfusion coupling. Oxygenation response to this manoeuvre is heterogeneous and derecruitment of dependent lung has not been elucidated. Nine pigs (32.2 ± 1.2 kg) were sedated and mechanically ventilated. Mono-lateral right-sided pneumonia was induced with intrabronchial challenge of *Pseudomonas aeruginosa*. After 24 h, lungs were recruited and the animals were randomly positioned on right or left side. After 3 h of lateral positioning, the animals were placed supine; another recruitment manoeuvre was performed, and the effects of contralateral decubitus were assessed. Primary outcome was lung ultrasound score (LUS) of the dependent lung after 3-h lateral positioning. LUS of the left non-infected lung worsened while positioned in left-lateral position (from 1.33 ± 1.73 at baseline to 6.78 ± 4.49; *p* = 0.005). LUS of the right-infected lung improved when placed upward (9.22 ± 2.73 to 6.67 ± 3.24; *p* = 0.09), but worsened in right-lateral position (7.78 ± 2.86 to 13.33 ± 3.08; *p* < 0.001). PaO_2_/FiO_2_ improved in the left-lateral position (*p* = 0.005). In an animal model of right-lung pneumonia, left-lateral decubitus improved oxygenation, but collapsed the healthy lung. Right-lateral orientation further collapsed the diseased lung. Our data raise potential clinical concerns for the use of lateral position in mono-lateral pneumonia.

## Introduction

Pneumonia is one of the most frequent causes of intensive-care unit (ICU) admission and mortality^1^. In the vast majority of cases, pneumonia commonly affects a single lobe, but the infection could broaden to an entire lung or ultimately lead to acute respiratory distress syndrome (ARDS)^2^. Severe mono-lateral pneumonia is one of the most challenging situations, since invasive mechanical ventilation (MV) is often needed to maintain adequate gas exchange, while substantial imbalance in pulmonary mechanics ensues between the healthy highly compliant lung and the stiff diseased lung. In the most severe cases, clinicians often place the patient onto lateral decubitus, with the healthy lung dependent, to improve ventilation-perfusion coupling and oxygenation^3^. Concurrently, recruitment of the non-dependent infected lung is desirable, and redistribution of ventilation has been observed^4–7^.

In the seminal study by Zack and colleagues^8^ the role of body position on arterial blood gas was appraised in patients with or without mono-lateral lung diseases. In those who presented mono-lateral disease, significant increase in arterial partial pressure of oxygen (PaO_2_) was demonstrated, while lying on the healthy side. Further corroboration of these findings are largely available^9–14^. In particular, through multiple inert-gas elimination technique, it was demonstrated that the aforementioned improvement in oxygenation was due to either reduction in intrapulmonary shunt or improvement in ventilation-perfusion mismatch (V_A_/Q)^9^. Since then, the use of lateral decubitus during mono-lateral lung disease has been employed to reduce the rate of tracheal intubation^11^ or ameliorate the respiratory stability. However, previous publications reported highly heterogeneous results; thus, benefits and safety of the lateral-positioning are still uncertain^13,14^. Indeed, when the healthy lung is placed downwards, the weight of the contralateral lung could theoretically promote pulmonary derecruitment, further complicated by the imposing heart weight.

Accordingly, we hypothesised that in an animal model of mono-lateral pneumonia, lateral position could be associated with deleterious effects associated with the collapse of the dependent lung. We aimed at comprehensively evaluate the effects of lateral position on lung aeration, through lung ultrasound, and gas exchange and pulmonary mechanics.

## Results

All nine animals (32.2 ± 1.2 kg) completed the study protocol. Before commencement of the study, the animals were ventilated as follows: V_T_ 8.1 [7.9–8.1] ml/kg, RR 28 [24–28] breaths per minute, FiO_2_ (%) 60 [55–60], PEEP 10 [8–10] cm H_2_O. Upon diagnosis of pneumonia, right-lung BAL resulted positive in 100% of the subjects, yielding *P. aeruginosa* concentration of 3.98 ± 0.18 log CFU/ml.

### Primary outcome

#### Lung ultrasound

**Figure 1:**
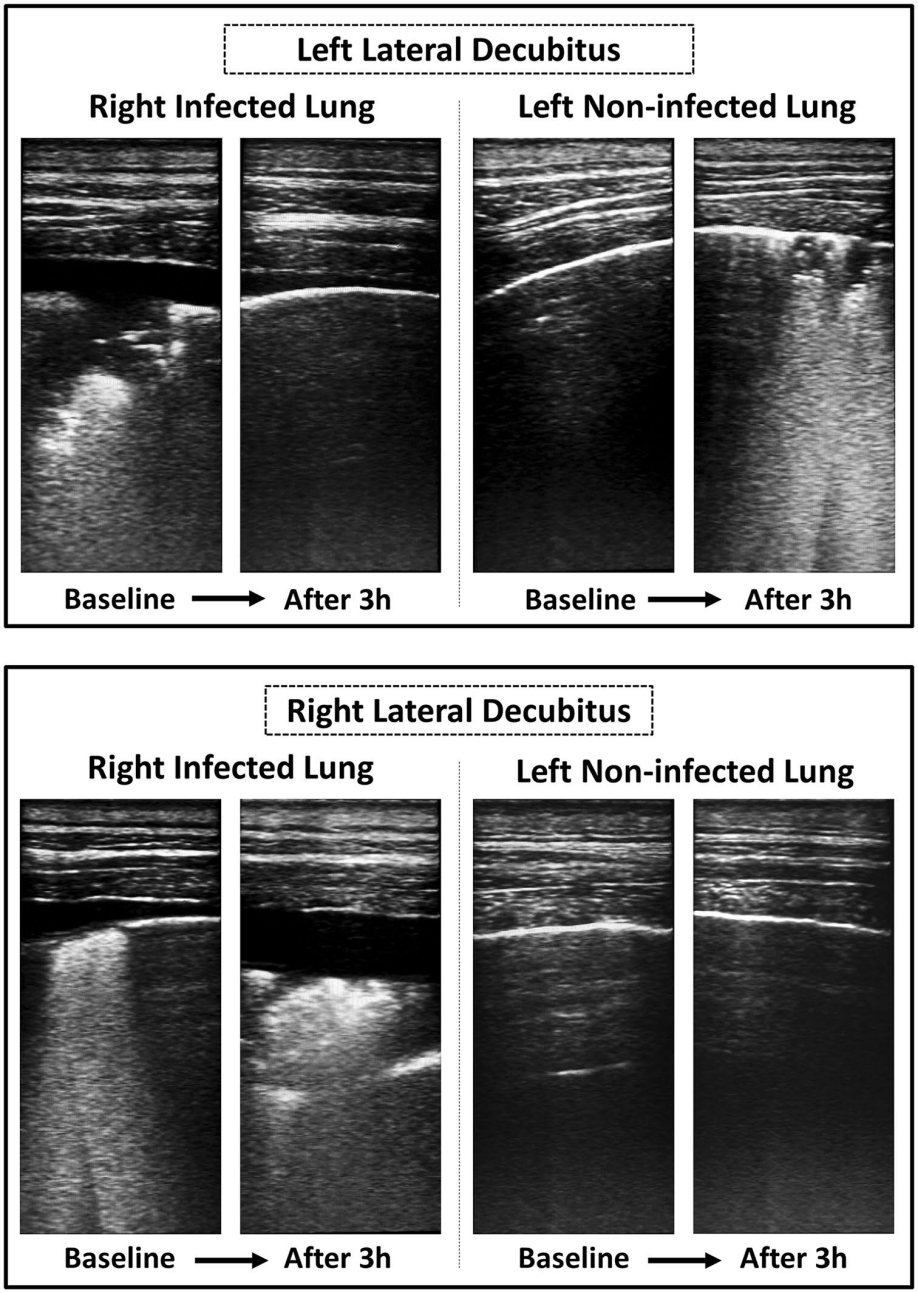
Lung Ultrasound images. In the upper section, a decrease in lung aeration is observed in the non-infected lung when dependent. In contrast, the infected lung (non-dependent) shows some re-aeration. In the lower section, lung ultrasound worsens further for the infected dependent lung.

**Figure 2:**
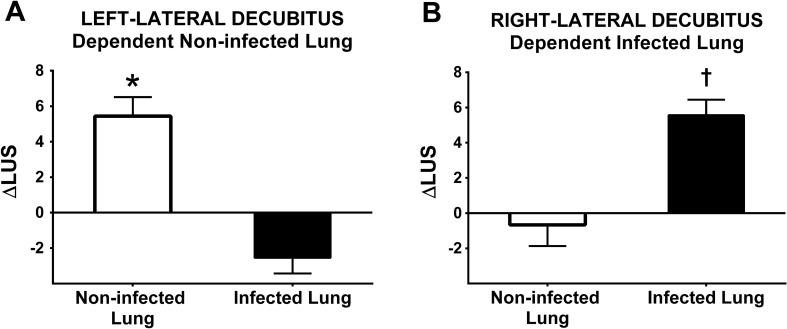
Lung Ultrasound Score modification (∆LUS) from baseline to 3 h for both positions. (**A**) In left-lateral position, causing the non-infected lung to be in a dependent position, LUS of the non-infected lung increased significantly, while LUS of the infected lung improved of a lesser extent. *p = 0.005; (**B**) In right-lateral position, causing the infected lung to be in a dependent position, LUS of the dependent lung showed marked worsening, while no relevant changes were observed in LUS of the non-infected lung. ^†^*p* < 0.001. ∆LUS: Delta Lung Ultrasound Score.

Figure 1 depicts the changes in lung ultrasound over time. LUS difference from baseline to 3 h (∆LUS) of the non-infected lung (left lung) worsened significantly during left-lateral position (+ 5.44 ± 1.07; *p* = 0.005), whereas it remained stable when the animal was placed in the right-lateral position (− 0.67 ± 1.20; *p* = 0.55), as summarised in Fig. 2. As for the infected right-lung, although a trend towards decreasing LUS score was observed after 3 h of decubitus on the left side (− 2.55 ± 0.88; *p* = 0.09), ∆LUS significantly worsened in right-lateral position (+ 5.56 ± 0.88; *p* < 0.001). Further LUS data analysis are reported in Table S1 and Fig. S2 of the Supplementary Content. In addition, the dynamics of lung aeration throughout the protocol are summarised in the enclosed lung ultrasound video (Lung Ultrasound Clip.mp4).

### Secondary outcomes

#### Gas exchange

**Figure 3:**
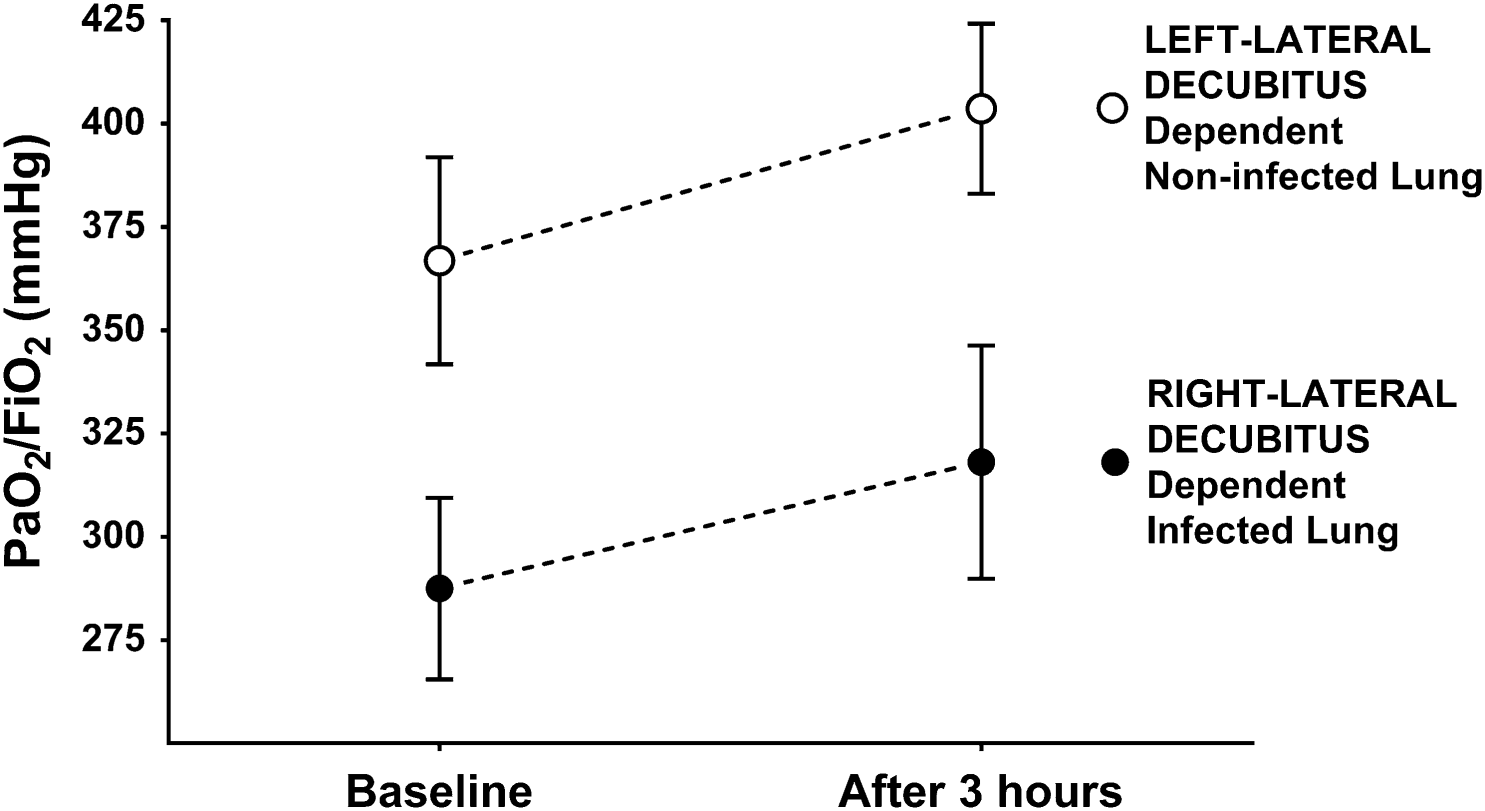
PaO_2_/FiO_2_ at baseline and after 3 h. PaO_2_/FiO_2_: arterial partial pressure of oxygen/inspiratory fraction of oxygen ratio.

**Figure 4:**
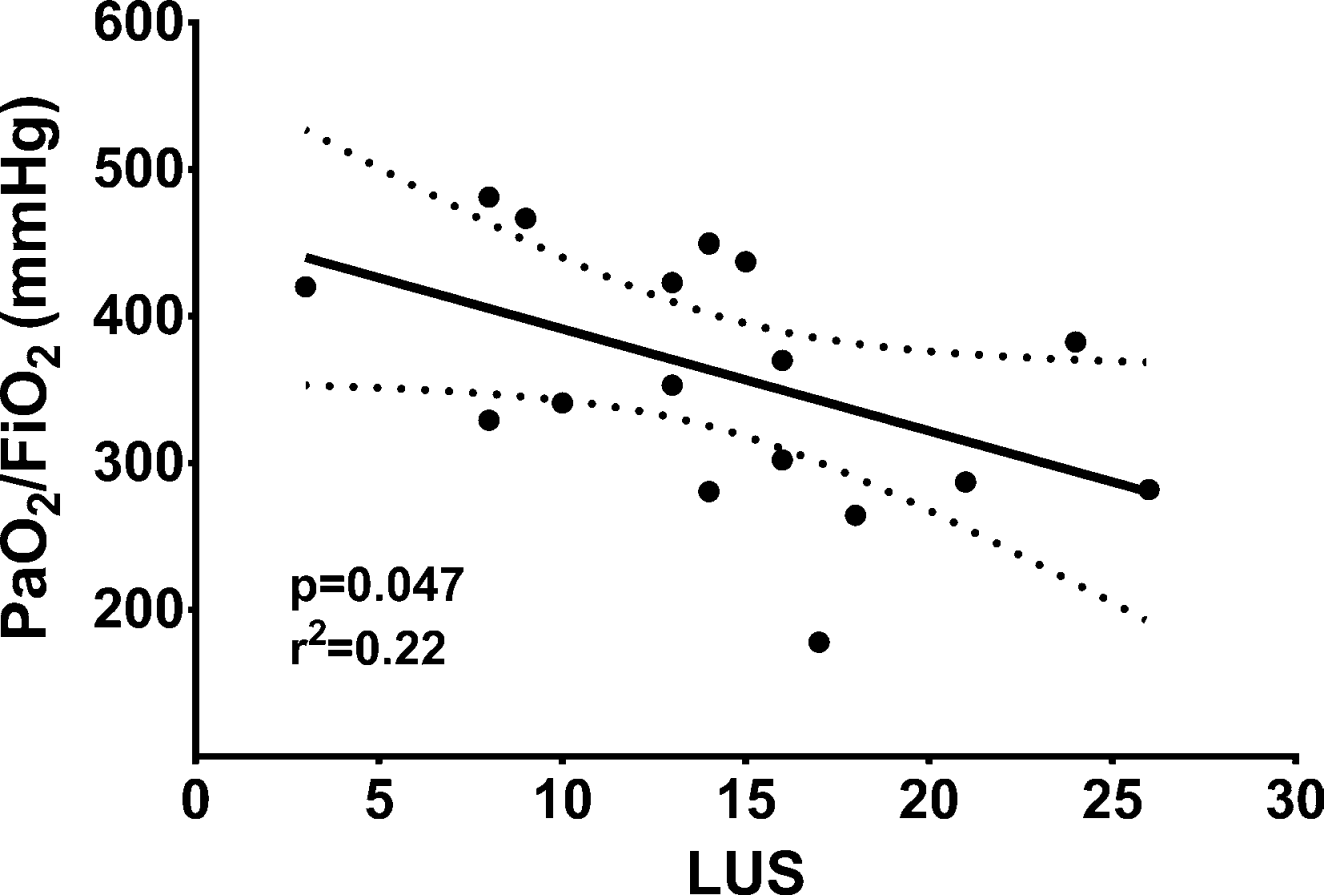
PaO_2_/FiO_2_ and LUS linear regression analysis at 3 h. Linear regression analysis between ratio of arterial partial pressure of oxygen and inspiratory fraction of oxygen (PaO_2_/FiO_2_) and lung ultrasound score (LUS) yielded a statistically significant correlation (*p* = 0.047; r^2^ = 0.22). Dotted lines show the 95% confidence interval of the best-fit line.

Table 1 displays the main clinical results. At baseline, and after 3 h, PaO_2_/FiO_2_ was consistently higher when the non-infected left lung was dependent (Fig. 3, *p* = 0.005). Indeed, the mean difference between the two positions was 79.35 ± 22.39 mmHg at baseline and 85.55 ± 22.19 mmHg after 3 h. Only one animal showed a decrease in PaO_2_/FiO_2_ from baseline during left-lateral position. PaO_2_/FiO_2_ slightly increased—although not significantly—throughout the 3 h of observation, irrespective of which position the animals were lying on. Furthermore, PaO_2_/FiO_2_ was associated with LUS at 3 h (*p* = 0.047; r^2^ = 0.22), as illustrated in Fig. 4. In contrast, partial pressure of carbon dioxide did not vary during changes in body position.

**Table 1:** Main study results. Data (mean ± SD) are reported per lateral-position and time of assessment.

Variables	Right-lateral decubitus dependent infected lung		Left-lateral decubitus dependent non-infected lung		*p* value		
	Baseline	After 3 h	Baseline	After 3 h	Time	Lateral position	Time* lateral position
PaO_2_/FiO_2_ (mmHg)	287.48 ± 65.94	318.06 ± 84.76	366.83 ± 75.25	403.61 ± 61.74	0.09	0.005	0.77
PaCO_2_ (mmHg)	36.97 ± 2.14	37.17 ± 4.98	37.30 ± 5.68	36.37 ± 5.04	0.68	0.88	0.39
MAP (mmHg)	76.55 ± 12.01	74.66 ± 5.07	68.77 ± 4.60	79.44 ± 9.74	0.11	0.69	0.049
mPAP (mmHg)	21.00 ± 2.65	20.44 ± 2.60	19.55 ± 2.65	19.88 ± 2.98	0.82	0.18	0.09
PVR (dyne.sec.cm^-5^)	286.56 ± 77.80	258.81 ± 84.79	273.43 ± 55.51	270.42 ± 63.24	0.43	0.96	0.18
CO (l/min)	2.56 ± 0.61	2.88 ± 0.83	2.52 ± 0.47	2.91 ± 0.97	0.11	0.97	0.82
Shunt (%)	29.00 ± 3.73	28.85 ± 5.32	25.84 ± 5.01	28.60 ± 5.53	0.31	0.29	0.10
E_RS_ (cmH_2_O/l)	35.69 ± 3.33	36.61 ± 3.02	36.94 ± 4.74	38.66 ± 6.13	0.18	0.20	0.43
E_L_ (cmH_2_O/l)	29.46 ± 4.53	28.88 ± 3.54	29.46 ± 3.89	31.06 ± 4.73	0.60	0.48	0.14
E_CW_ (cmH_2_O/l)	6.73 ± 1.79	8.25 ± 2.17	8.54 ± 1.65	8.71 ± 1.93	0.16	0.20	0.24
∆P_L_ (cmH_2_O)	7.56 ± 1.22	7.39 ± 0.93	7.55 ± 1.05	7.96 ± 1.28	0.62	0.48	0.14
DP_AW_ (cmH_2_O)	9.16 ± 0.94	9.39 ± 0.73	9.48 ± 1.29	9.92 ± 1.58	0.20	0.20	0.42

PaO_2_/FiO_2_, Arterial partial pressure of oxygen/Inspiratory fraction of oxygen ratio; PaCO_2_, Arterial partial pressure of carbon dioxide; MAP, mean arterial pressure; mPAP, mean pulmonary arterial pressure; PVR, pulmonary vascular resistance; CO, cardiac output; E_RS_, respiratory system elastance; E_L_, lung elastance; E_CW_, chest wall elastance; ∆P_L_, transpulmonary pressure; DP_AW_, driving pressure of the airway.

#### Pulmonary mechanics and hemodynamics

A minor increase in lung elastance (E_L_) was recorded during left-lateral position (infected lung upward), while it marginally decreased in right-lateral position (Supplementary Fig. S3); both changes were shy of statistical significance. Indeed, as reported in Table 1, there were no significant changes in pulmonary mechanics throughout the study. Finally, apart from a slight improvement in mean arterial pressure in the left-lateral position (infected lung upward), no relevant hemodynamic changes were observed.

## Discussion

In animals with severe mono-lateral right pneumonia, we comprehensively evaluated aeration dynamics during change in side of lateral position. In particular, our study is the first to confirm that irrespective of improvements in oxygenation, loss of air content is evident when the healthy lung is positioned in gravity-unfavourable position.

During severe mono-lateral lung disease, improvement in oxygenation has consistently driven the use of lateral decubitus on the healthy side^8–11^, irrespective of the surrounding controversy^15,16^, and lack of robust evidence on lung recruitment/derecruitment during lateral position. In particular, in clinical settings, lung aeration/de-aeration is not routinely monitored, especially during lateral position. In the last decade, there has been a surge of interest in lung ultrasound among critical care physicians^17^ due to the possibility of reliable monitoring the dynamics of lung recruitment/derecruitment in response to interventions^18,19^, with a few controversial arguments regarding potential limitations^20^.

To the best of our knowledge, our experimental study is the first to demonstrate that in a model of mono-lateral pneumonia, lateral position causes de-recruitment of the dependent lung. This was clearly corroborated by an increase in LUS, during the short 3-h follow-up period. Conversely, in line with previous reports, the infected lung benefited from being placed upward, as showed by the improvement in LUS score^6^. As expected, lung derecruitment is driven by the force of gravity, which unfavourably wields lung and heart weight to the dependent lung, potentially overcoming end-expiratory alveolar pressures. This effect could be even more pronounced when the left lung is dependent, due to its smaller volume vs. the right one^21^. Loss of aeration in the healthy dependent lung might then account for the heterogeneity of response observed in clinical studies^8,12^, where the use of lateral position on this lung is not always followed by an improvement in oxygenation. In this context, the application of PEEP is crucial to avoid these potential complications^18^. Bsut at times it could be insufficient to avoid de-aeration, as also hypothesised in an experimental study on rats^22^.

The influence of body position on gas distribution is well known in ARDS, where prone positioning is commonly indicated as a rescue therapy in severely-ill patients. In prone position, the anatomical and functional distribution of lung ventilation is remarkably modified^23^, and as a result oxygenation, pulmonary mechanics and mortality improved^24^. Unlike the mechanisms responsible for oxygenation improvement during prone position, in which the redistribution of ventilation leads to recruitment of the dorsal zones exceeding de-recruitment of the ventral ones^25^, the most probable mechanism in lateral positioning is amelioration of V_A_/Q mismatch and reduction of pulmonary shunt, caused by the redistribution of blood flow to more ventilated areas^9^. Furthermore, as demonstrated by the lack of changes in PaCO_2_, lateral position in mono-lateral pneumonia marginally affects physiological dead space.

Interestingly, in our study the improvement in arterial oxygenation was observed, irrespective of dependent lung derecruitment, in all animals but one. This could have been promoted by recruitment of the non-dependent sick lung, as observed during postural changes in mechanically ventilated children by Tusman and collaborators^6^. The amelioration in blood oxygenation in our study was sustained during the three-hour period of assessment, but it should be also emphasised that pulmonary shunt—with the healthy lung down—showed a slight worsening trend of approximately 3% throughout the follow up period.

In contrast with previous studies^4,16^, pulmonary mechanics did not worsen as a result of change in position. In particular, we found a driving pressure in the range of 9 cm H_2_O suggesting low risk for ventilator-induced lung injury in this model of mono-lateral pneumonia. Nevertheless, pulmonary mechanics could be unspecific in monitoring lung injury in this model, and clinical data are needed to evaluate potential association of lateral position with lung injury and clinically-relevant outcomes^26^. Similarly, in line with previous data^27^, hemodynamic measurements did not differ between positions. Yet, it should be taken into account that clinical evidence has shown that mobilization of severely-ill patients can drastically impair hemodynamic stability^16,28^, hence risks should be pondered upon indication.

Our *in-vivo* study provides some original insights that hold clinical promise. First, in clinical settings patients with mono-lateral pneumonia may be kept in the lateral position up to several hours^11,12,16^. Theoretically, a progressive impairment of lung aeration and oedema accumulation over time could be expected, but linear decrease in loss of aeration could not be entirely inferred by our findings and should be corroborated in future experiments also taking into account various ventilatory settings. Our findings call for translational clinical studies that could further address these key aspects and potentially identify valuable markers to be monitored in order to guide safe duration for lateral position. Second, although we did not focus on displacement of biofluids in our settings, which was previously studied in small animals^22^, during prolonged lateral positioning infected biofluids seepage into the dependent lung could further compromise lung function and future comprehensive clinical research needs to address these incompletely characterised pathophysiologic mechanisms.

A few limitations of our study should be discussed. First, we used a model of right-lung pneumonia, hence our findings should be carefully extrapolated to left-lateral pneumonia. Nevertheless, our observations are in lines with previous reports in patients with left or right diseased lungs^8–12^. Second, we only monitored lung derecruitment for 3 h. Lastly, although we conducted inclusive sample size analysis, our population was rather limited in size.

## Methods

The study was conducted at the Division of Animal Experimentation, Department of Pulmonary and Critical Care Medicine, Hospital Clínic (Barcelona, Spain) in accordance with the European 2010/63/UE and Spanish RD 53/2013 regulations related to the Guide for the Care and Use of Laboratory Animals^29^. The study was approved by the Animal Experimentation Ethics Committee of the University of Barcelona (571/16) on 30 November 2016.

We designed a prospective cross-over randomised experimental study, primarily aimed at evaluating lung aeration dynamics, specifically in the healthy lung, in mechanically-ventilated pigs, with severe mono-lateral pneumonia. Secondary endpoints were variations in gas exchange, pulmonary mechanics and hemodynamic parameters.

### Model of mono-lateral right pneumonia

This study was carried out in nine Large White—Landrace female pigs (Specipig, Barcelona, Spain). Each animal was challenged immediately following preparation and stabilization. Fifteen ml of 10^7^ colony-forming unit (CFU)/ml of a log-phase culture of clinical *Pseudomonas aeruginosa* strain was instilled, through bronchoscopy, into the right upper, middle and lower lobe^30^. Upon instillation, and 20 h thereafter, the animals were kept in lateral-right decubitus to develop right-lung pneumonia. After 24 h of MV, diagnosis of pneumonia was confirmed based on clinical and laboratory data (Supplementary Content, “Methods”). Moreover, bronchoalveolar lavage (BAL) was performed to confirm the diagnosis of mono-lateral pneumonia.

### Study protocol

**Figure 5:**
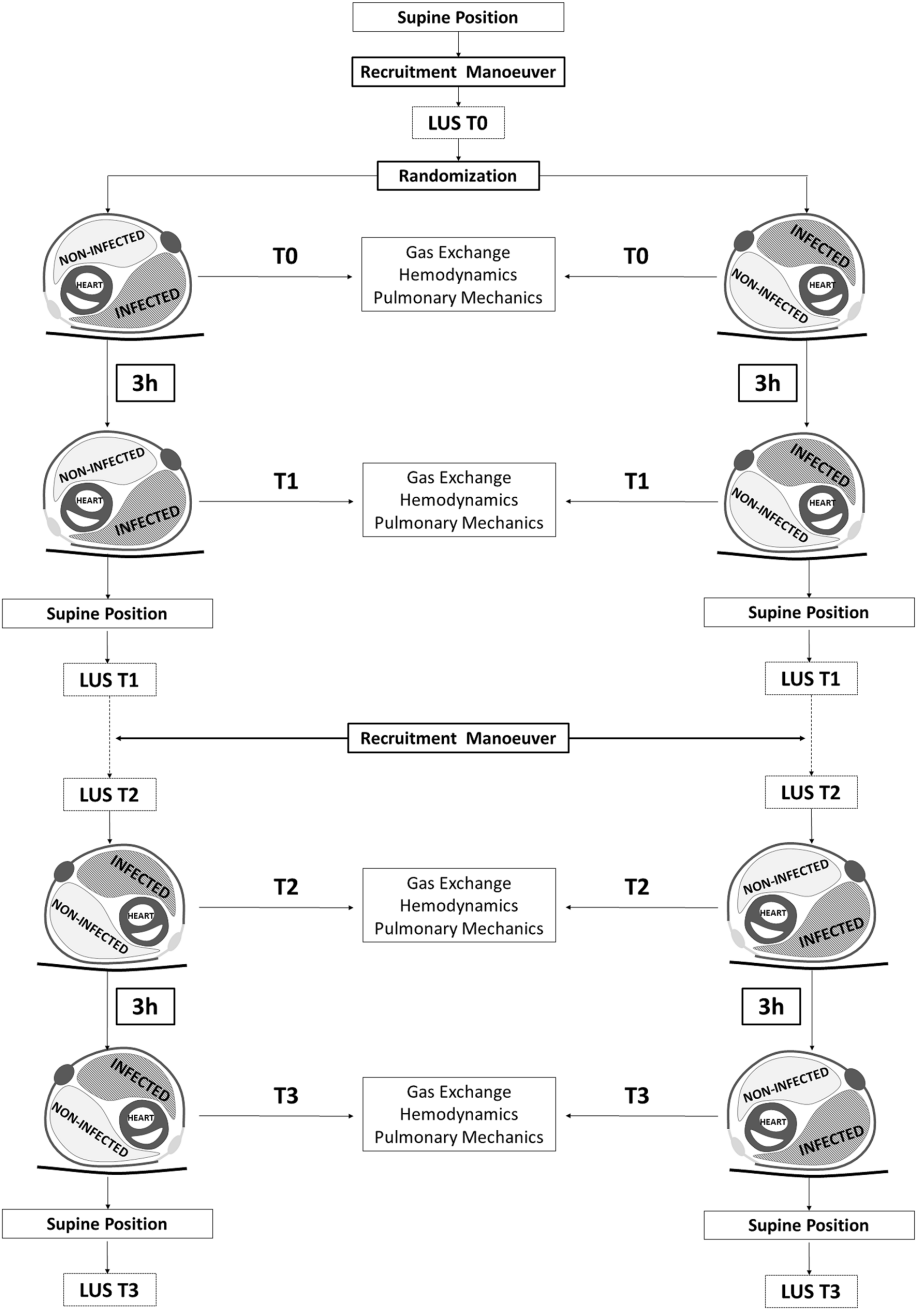
Flowchart. RM, recruitment manoeuvre; LUS, lung ultrasound score. Realized with Microsoft Word 2010 (https://www.microsoft.com/) and Adobe Photoshop CC 2019 (https://adobe.com).

Figure 5 displays the study protocol. Also, a thorough description of animal preparation, instrumentation and monitoring can be found in the Supplementary Content (“Methods”). Of note, tidal volume (V_T_), positive end-expiratory pressure (PEEP), respiratory rate (RR), and inspiratory fraction of oxygen (FiO_2_) were kept constant throughout the study. The animal was first placed in supine position and a recruitment manoeuvre (RM) was performed as detailed in the Supplementary Content (“Methods”). At the end of RM, the previous ventilator settings were restored. Baseline lung ultrasound (LUS) was performed (T0), then the animal was randomly placed onto the first lateral side (Supplementary Content, Randomization List). After 15-min of stabilization in lateral position, baseline hemodynamic parameters, gas exchange, and lung mechanics were measured. After 3 h, the aforementioned assessments were repeated (T1). Then, the animal was placed supine and LUS at T1 was performed. After LUS monitoring, RM was repeated and then another LUS evaluation was completed (T2). The animal was then positioned on the contralateral side, and aforementioned assessments were performed at baseline (T2) and after 3 h (T3). Lastly, the animal was placed again in supine position and LUS T3 was recorded.

### Lung ultrasound

Lung ultrasound was performed by trained intensive care physicians with more than 5-year experience in lung ultrasound imaging. A linear probe (MicroMaxx L25e/13–6 MHz, Sonosite Inc., Bothell, WA, USA) was employed to evaluate right and left ventral, intermediate and dorsal thoracic fields. Each field was divided into a cranial and a caudal subsection, as depicted in Fig. 5 of the Supplementary Content. Recorded lung ultrasound images were subsequently evaluated by one operator, blinded to the study interventions (time and position of the animal) and to randomization order, who computed LUS of both lungs using a four-grade scoring system^31^: 0, normal lung ultrasound (multiple horizontal A-lines); 1, at least three separated B-lines; 2, coalescent B-lines; 3, consolidation. Per each time of assessment, we computed the total LUS pulmonary score, and right and left lung scores. Thus, LUS score per each hemithorax ranged from 0 to 18; the higher the score, the higher was the loss in pulmonary aeration of a single lung.

### Respiratory and hemodynamic measurements

Airway pressure and respiratory flow rates were measured as previously reported^17^. For a detailed description of methods please see the Supplementary Content (Pulmonary Mechanics). Arterial and venous pressures were measured with disposable pressure transducers (TrueWave Pressure Transducer, Edwards Lifescience, Irvine, CA). Pulmonary arterial pressure, central venous pressure, pulmonary arterial wedge pressure, core blood temperature, and cardiac output were measured using a Swan–Ganz catheter (Swan-Ganz PAC, Edwards Lifesciences, Irvine, CA). The systemic and pulmonary vascular resistances and venous admixture were calculated using standard formulae^32^.

### Statistical analysis

Continuous variables are described as mean ± standard deviation (SD), or median [IQR] in case of non-parametric parameters. In order to evaluate the effects of time and position on this animal model, we used a restricted minimum likehood (REML) analysis for mixed models. A (co)variance structure was used to model the within-subjects errors and the Kenward-Roger approximations to estimate denominator degrees of freedom. For each continuous variable, the overall F test was assessed for significance. All post-hoc comparisons were adjusted through Bonferroni correction. A two-sided *p* value ≤ 0.05 was considered statistically significant. Pearson coefficients were used to compute correlation analyses. We finally conducted a sample size analysis, which is reported in the Supplementary Content. All statistical analyses were performed using SAS software (version 9.4; SAS Institute, Cary, NC).

### Ethics approval

See “Methods” section.

### Prior abstract presentation

An abstract from this study was presented at the 2018 European Society of Intensive Care Medicine (ESICM) congress in Paris, October 20th–24th.

## Supplementary information

Supplementary Video 1.

Supplementary Information 1.

## Data Availability

The datasets used and/or analysed during the current study are available from the corresponding author on reasonable request.

## Abbreviations

∆P_L_: Transpulmonary pressure
BAL: Bronchoalveolar lavage
CFU: Colony-forming unit
CO: Cardiac output
DP_RS_: Driving pressure of the respiratory system
E_CW_: Chest wall elastance
E_L_: Lung elastance
E_RS_: Respiratory system elastance
FiO_2_: Inspiratory fraction of oxygen
FRC: Functional residual capacity
ICU: Intensive care unit
LUS: Lung ultrasound score
MAP: Mean arterial pressure
mPAP: Mean pulmonary arterial pressure
MV: Mechanical ventilation
PaCO_2_: Arterial partial pressure of carbon dioxide
PaO_2_/FiO_2_: Arterial partial pressure of oxygen/inspiratory fraction of oxygen ratio
PaO_2_: Arterial partial pressure of oxygen
PCV: Pressure-controlled ventilation
PEEP: Positive end-expiratory pressure
RM: Recruitment manoeuvre
RR: Respiratory rate
V_A_/Q: Ventilation-perfusion ratio
VILI: Ventilation-induced lung injury
V_T_: Tidal volume
