# Supplementary material for: Lateral position during severe mono-lateral pneumonia: an experimental study
Source: Sci Rep. 2020 Nov 9;10:19372. doi: 10.1038/s41598-020-76216-w (PMC7653044; doi:10.1038/s41598-020-76216-w)

# Supplementary Content

**LATERAL POSITION DURING SEVERE MONO-LATERAL PNEUMONIA: AN EXPERIMENTAL STUDY**

Andrea Meli, Enric Barbeta Viñas, Denise Battaglini, Gianluigi Li Bassi, Hua Yang, Minlan Yang, Joaquim Bobi, Anna Motos, Laia Fernández-Barat, Davide Chiumello, Paolo Pelosi, and Antoni Torres

**Table of Contents**

**Additional Methods:**

Animal Model

*Instrumentation and initial ventilator settings……………………………..…………………………….p. 3*

*Diagnosis of pneumonia…………………………………………………………………………………….p. 3*

*Scheduled clinical assessments……………………………………………….……………………...…….p. 4*

*Recruitment Manoeuvres…………………………………………………………………………….…......p. 4*

*Pulmonary mechanics.………………………………………………………………………………………p. 4*

*Sample size analyses…………………………………………………………………………..…………….p. 5*

*Randomization List…………………………………………………………………………………………..p. 6*

*References…………………………………………………………………………………………………….p. 7*

**Additional Tables:**

*Table S1 – LUS values……………....……………………………………………………………….……..p. 8*

**Additional Figures:**

*Figure S1 - Schematised anatomy and LUS areas…………………..……………………….……........p. 9*

### *Figure S2 - LUS modification…………………………………….....…………………………….……...p. 10*

*Figure S3 - Respiratory system, lung, and chest wall elastance…….…….…………………………p. 11*

### Additional Methods

### *Animal Model – Instrumentation and initial ventilator settings*

This study was carried out in 9 Large White-Landrace female pigs (Specipig, Barcelona, Spain) undergoing mechanical ventilation. Animals were sedated with intramuscular ketamine (6 mg/kg), xylazine (3,5 mg/kg) and midazolam (0.15 mg/kg). Propofol (2 – 2.5 mg/kg) was given to induce anaesthesia and to tracheally intubate the animals with a 7.5 mm I.D tracheal tube (ETT) (Hi-Lo^®^, Covidien, Boulder, CO). Following intubation, pigs were ventilated through a SERVO-i mechanical ventilator (Maquet, Wayne, NJ. USA). Ventilatory parameters were initially set as follows: volume-control, tidal volume (V_T_) 8 mg/kg, pressure trigger sensitivity of -2 cm H_2_O, inspiratory fraction of oxygen (FiO_2_) 0.4, duty cycle 0.33, inspiratory rise time 5%, inspiratory pause 10%, PEEP 4 cm H_2_O and respiratory rate (RR) adjusted to maintain normocapnia. Inspiratory gases were conditioned through a heated humidifier along the inspiratory limb (MR850, Fisher & Paykel, Auckland, New Zealand). Throughout the study, internal ETT cuff pressure was maintained at 28 cm H_2_0. A nasogastric cuffed catheter (CareFusion, Yorba Linda, CA, USA) was placed in order to assess oesophageal pressure. Midazolam and fentanyl were administered to ensure absence of response to painful stimulation, as already reported in previous works [1]. Boluses of 2 mg/kg of propofol were administered as needed. Ultrasound-guided cannulation of the femoral artery was performed for systemic arterial pressure monitoring and collection of blood samples. Ultrasound-guided cannulation of the jugular vein was performed to insert an 8-Fr introducer and a 7-Fr Swan-Ganz catheter (Swan-Ganz PAC, Edwards Lifesciences, Irvine, CA) for advanced hemodynamic monitoring. A no. 12 Foley catheter was introduced into the urinary bladder through the urethra.

*Animal Model – Diagnosis of pneumonia*

After 24 hours of MV (approximately 20 hours from bacterial challenge), diagnosis of pneumonia was confirmed based on the following variables: a decline in arterial partial pressure of oxygen/inspiratory fraction of oxygen ratio (PaO_2_/FiO_2_) ≤100 mmHg from baseline plus one of the following signs of infection: a temperature of ≥39.5 °C, leukocytosis of ≥20 x 10^9^ cells/l and purulent secretions. Right lung bronchoalveolar lavage (BAL) was performed to confirm the diagnosis of mono-lateral pneumonia.

*Animal Model - Scheduled clinical assessments*

Fluid balance was maintained through infusion of lactate Ringer's and 0.9% NaCl solutions. In order to prevent pneumonia caused by endogenous oropharyngeal flora, 1 g of ceftriaxone was administered intravenously 30 min before intubation and then 50 mg/kg every 12 hours for the entire duration of the study. Every 6 hours arterial and mixed venous blood gases, hemodynamics, urine output and ventilatory settings were assessed. Every 24 hours pulmonary mechanics were assessed. Additionally, every 12 hours, complete blood count, biochemistry and coagulation studies were carried out and reviewed.

*Animal Model – Recruitment Manoeuvres*

Before T0 and T2 assessments, with the animal in supine position, two identical recruitment manoeuvres were carried out as follows: pressure-controlled ventilation (PCV), respiratory rate (RR) 10/min and I:E ratio of 1:1. Recruitment started with PEEP of 15 cm H_2_O and driving pressure of 15 cm H_2_O. These parameters were maintained for 1 min; then, PEEP was increased to 20 cm H_2_O for 1 min and then to 25 cm H_2_O for 2 min, with the other parameters unchanged. At the end of RM, the previous ventilator settings were restored.

*Animal Model – Pulmonary mechanics*

Pulmonary mechanics were computed through the analysis of ventilatory flow, airway and oesophageal pressures waveforms. The correct position of the oesophageal catheter was confirmed as detailed in previous publication [2]. Flow and pressure signals were recorded on a personal computer for subsequent analysis with dedicated software (Colligo; Elekton, Milan, Italy; www.elekton.it). Tidal volumes were obtained by mathematical integration of the measured flow signal. Static elastances of the respiratory system (lung and chest wall) were calculated through the rapid occlusion method using standard formulae [3]. Driving pressure of the respiratory system (DP_AW_) was calculated through the formula: DP_AW_ = P_PLAT_ - PEEP, where P_PLAT_ is the plateau airway pressure obtained with 4-sec rapid occlusion method [4]. Transpulmonary pressure (∆P_L_) was calculated as follows: ∆P_L_ = (P_PLAT_ – PEEP) – (Pes_EI_ – Pes_EE_), where Pes_EI_ is the end-inspiratory oesophageal pressure (or oesophageal plateau pressure) and Pes_EE_ in the end-expiratory oesophageal pressure [5].

*Sample size analysis*

Our sample size analysis was based on the analysis of lung ultrasound score. In order to calculate such score, we evaluated right or left ventral, intermediate and dorsal thoracic fields. Each field was divided into a cranial and a caudal subsection. We applied a four-grade scoring system: 0, normal lung ultrasound (multiple horizontal A-lines); 1, at least three separated B-lines; 2, coalescent B-lines; 3, consolidation [6]. Per each time of assessment, we computed the total LUS pulmonary score, and right and left lung scores. Thus, LUS score per each hemithorax ranged from 0 to 18; the higher the score, the higher was the loss in pulmonary aeration of a single lung. Thus, to demonstrate whether the healthy lung could collapse while the animals was in the left lateral side, we assumed that the LUS score at baseline were 4 with an increase up to 8 after 3-hour lateral positioning, with a standard deviation ± 2.5. For an assumed effect size of 1.6, 2.49 of the two-tailed paired t-test, a desired statistical power of 90%, we would have needed a sample size of 7 pigs to demonstrate changes in LUS score between timepoints in the left healthy lung.

*Animal Model - Randomization List*

| Block identifier | Block size | Sequence within block | Treatment |
| --- | --- | --- | --- |
| 1 | 9 | 1 | R |
| 1 | 9 | 2 | L |
| 1 | 9 | 3 | L |
| 1 | 9 | 4 | R |
| 1 | 9 | 5 | L |
| 1 | 9 | 6 | L |
| 1 | 9 | 7 | R |
| 1 | 9 | 8 | R |
| 1 | 9 | 9 | L |

**Randomization list.** Treatment column depicts the first side adopted for lateral decubitus. R, right side; L, left side.

*References*

1. Li Bassi, G. *et al.* A novel porcine model of ventilator-associated pneumonia caused by oropharyngeal challenge with Pseudomonas aeruginosa. *Anesthesiology* **120**, 1205–15 (2014).

2. Baydur, A., Behrakis, P. K., Zin, W. A., Jaeger, M. & Milic-Emili, J. A simple method for assessing the validity of the esophageal balloon technique. *Am. Rev. Respir. Dis.* **126**, 788–91 (1982).

3. Akoumianaki, E. *et al.* The application of esophageal pressure measurement in patients with respiratory failure. *Am. J. Respir. Crit. Care Med.* **189**, 520–531 (2014).

4. Amato, M. B. P. *et al.* Driving Pressure and Survival in the Acute Respiratory Distress Syndrome. *N. Engl. J. Med.* **372**, 747–755 (2015).

5. Cortes-Puentes, G. A. *et al.* Impact of chest wall modifications and lung injury on the correspondence between airway and transpulmonary driving pressures. *Crit. Care Med.* **43**, e287–e295 (2015).

6. Bouhemad, B., Mongodi, S., Via, G. & Rouquette, I. Ultrasound for “ Lung Monitoring ” of Ventilated Patients. *Anesthesiology* **122**, 437–447 (2015).

*Additional Tables*

*Table S1.*

| LUS | Right-Lateral Decubitus  Dependent Infected Lung | | |  | Left-Lateral Decubitus  Dependent Non-Infected Lung | | |  |
| --- | --- | --- | --- | --- | --- | --- | --- | --- |
|  | Baseline |  | After 3h | p value | Baseline |  | After 3h | p value |
| LUS  (non-infected lung) | 2.66±2.91 |  | 2.00±2.73 | p=0.55 | 1.33±1.73 |  | 6.78±4.49 | p=0.005 |
| LUS  (infected lung) | 7.78±2.86 |  | 13.33±3.08 | p<0.001 | 9.22±2.73 |  | 6.67±3.24 | p=0.09 |

**Table S1. LUS values.** The table shows mean ± SD of LUS per lateral position side and time of assessment. P-values are shown for each comparison. See Figure S2 for the graphic representation of this data. LUS: Lung Ultrasound Score.

Additional Figures

**Figure S1. Schematised anatomy and LUS areas**. A: the surface of the thorax was divided into right (sick lung) and left (healthy lung) areas. The animal was placed in supine position; B: the right hemithorax was divided into six scan areas, as described in the text. R, right; L, left. Modified with Adobe Photoshop CC 2019 (http://adobe.com).

*
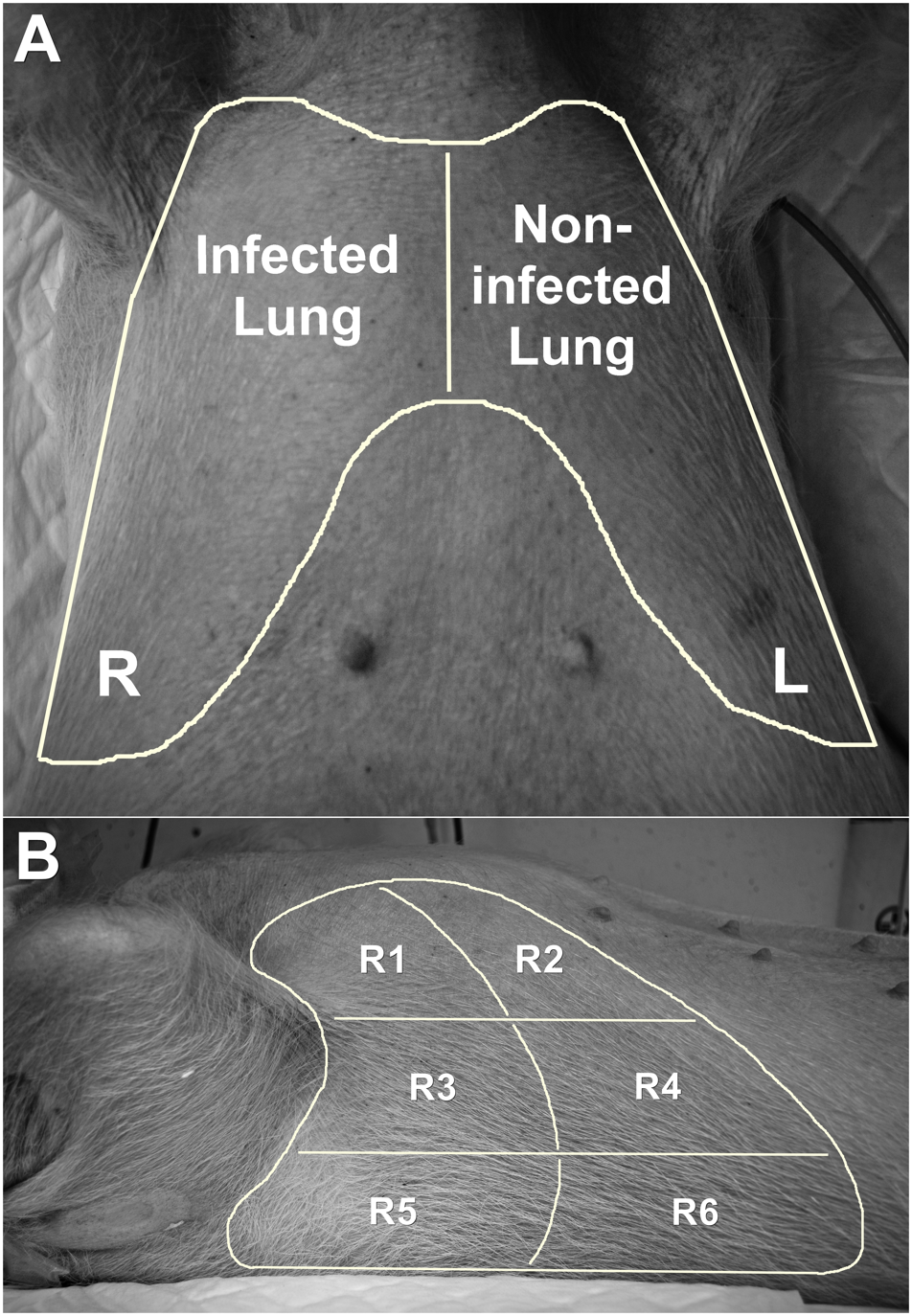
*

**Figure S2. LUS modification for each subject.** The graph shows modification of LUS of each lung at baseline and after 3 hours in the two positions (dependent healthy lung, A; dependent sick lung, B, respectively) for every subject. P-values for LUS change are shown on top of each graph subsection. LUS: Lung Ultrasound Score.


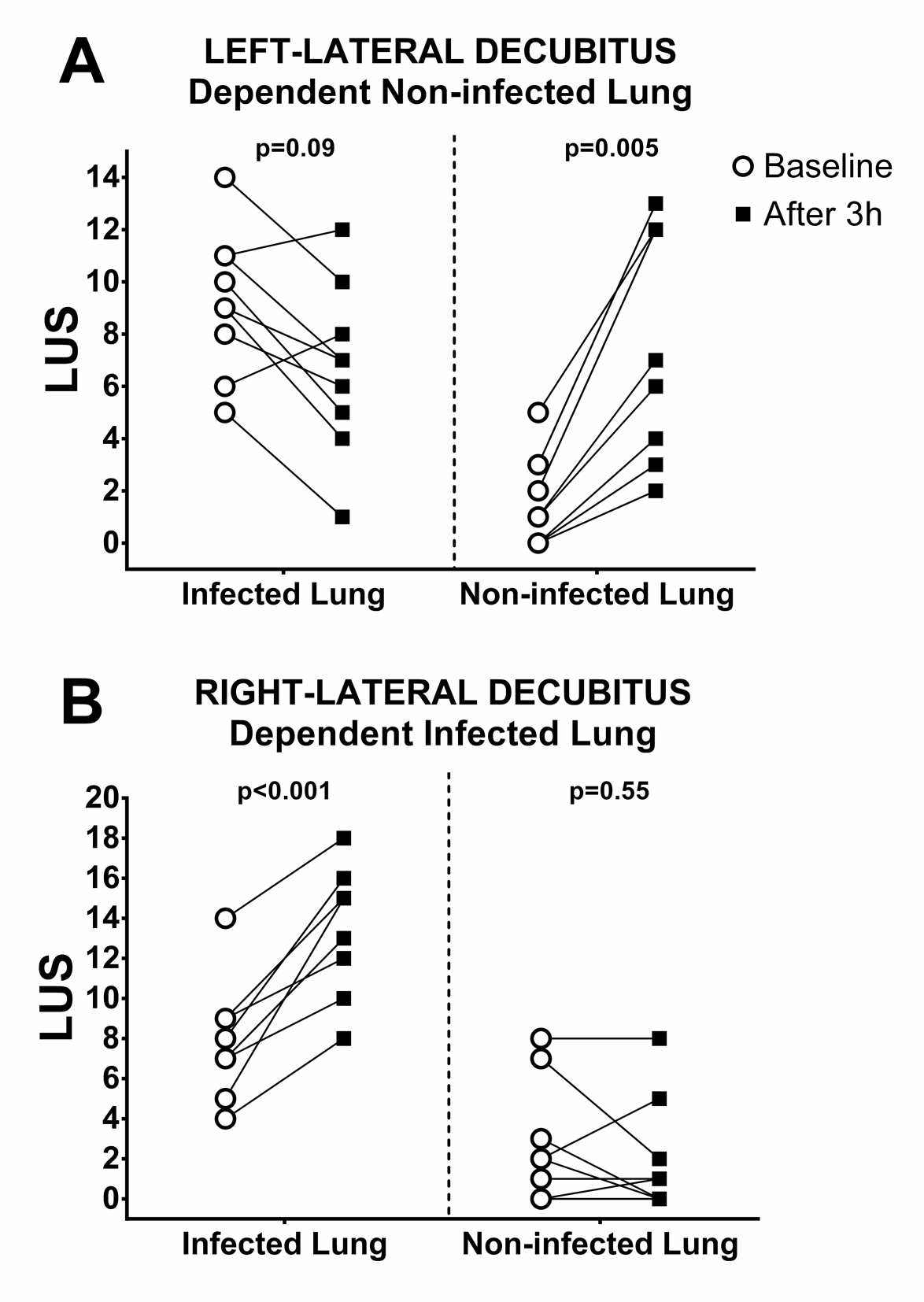


**Figure S3. Respiratory system, lung, and chest wall elastance.** During the 3-hours period, respiratory system elastance (E_RS_) slightly increased equally when the healthy or the sick lung were positioned downward. Similarly, a slight increase in lung elastance (E_L_) was found in the former case, while E_L_ decreased when the sick lung was placed downward. When lying on the infected lung, the animals yielded a minor increase in chest wall elastance (E_CW_). Nevertheless, these changes appeared shy of statistical significance.


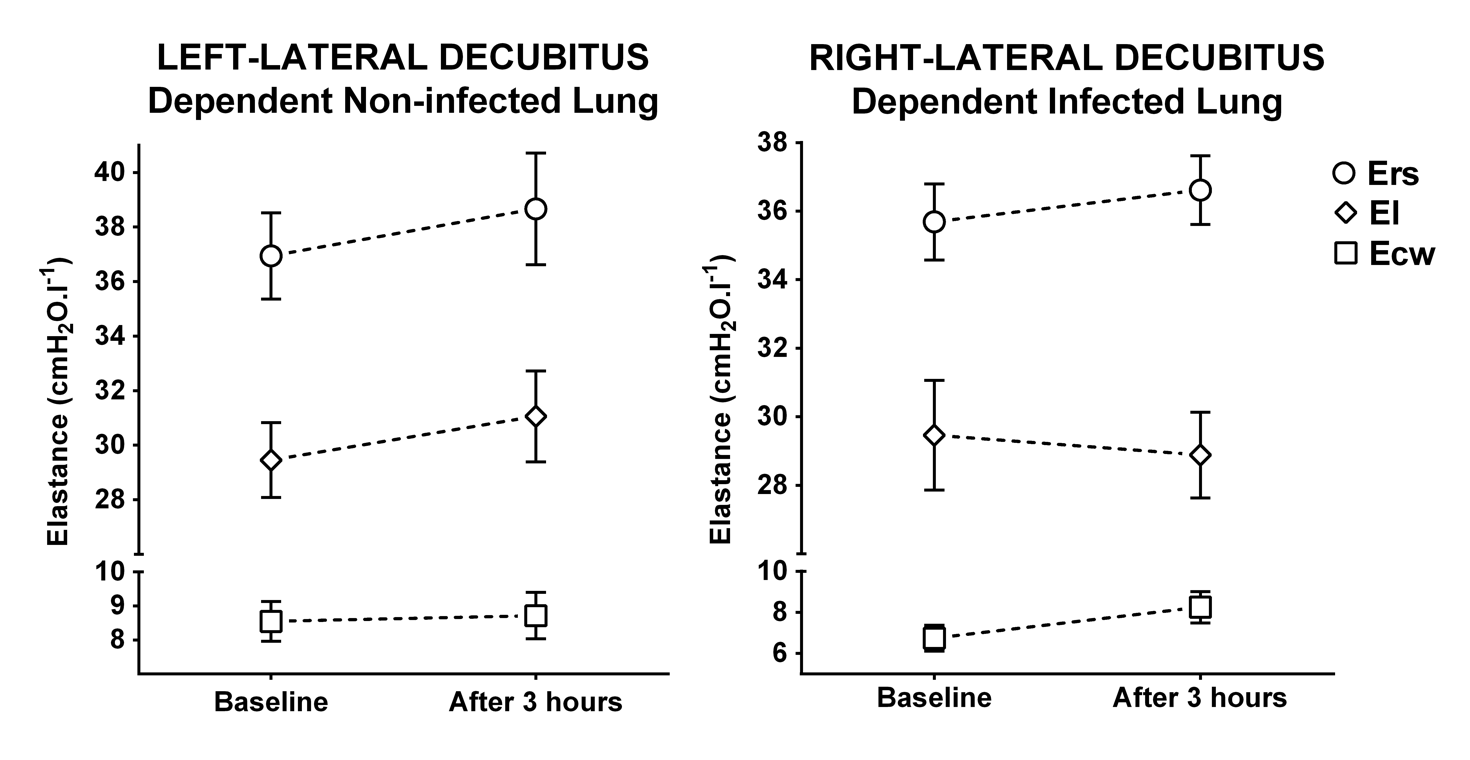

Supplement: Supplementary file 2 — Supplementary Information 1. [file 41598_2020_76216_MOESM2_ESM.docx]
